# Association of Metabolic Syndrome With Long-Term Cardiovascular Risks and All-Cause Mortality in Elderly Patients With Obstructive Sleep Apnea

**DOI:** 10.3389/fcvm.2021.813280

**Published:** 2022-02-07

**Authors:** Lin Liu, Xiaofeng Su, Zhe Zhao, Jiming Han, Jianhua Li, Weihao Xu, Zijun He, Yinghui Gao, Kaibing Chen, Libo Zhao, Yan Gao, Huanhuan Wang, JingJing Guo, Junling Lin, Tianzhi Li, Xiangqun Fang

**Affiliations:** ^1^Department of Pulmonary and Critical Care Medicine of the Second Medical Center and National Clinical Research Center for Geriatric Diseases, Chinese PLA General Hospital, Beijing, China; ^2^Medical College, Yan'an University, Yan'an, China; ^3^Cardiology Department of the Second Medical Center and National Clinical Research Center for Geriatric Diseases, Chinese PLA General Hospital, Beijing, China; ^4^PKU-UPenn Sleep Center, Peking University International Hospital, Beijing, China; ^5^Sleep Center, The Affiliated Hospital of Gansu University of Chinese Medicine, Lanzhou City, China; ^6^Department of General Practice, 960th Hospital of PLA, Jinan, China; ^7^Department of Pulmonary and Critical Care Medicine, Sleep Medicine Center, Peking University People's Hospital, Beijing, China; ^8^Department of Pulmonary and Critical Care Medicine, Beijing Chaoyang Hospital Affiliated to Capital Medical University, Beijing, China; ^9^The Second Medical Center and National Clinical Research Center for Geriatric Diseases, Chinese PLA General Hospital, Beijing, China

**Keywords:** obstructive sleep apnea, metabolic syndrome, elderly, major adverse cardiovascular events, mortality, cardiovascular disease

## Abstract

**Background:**

Evidence suggests that an increased risk of major adverse cardiac events (MACE) and all-cause mortality is associated with obstructive sleep apnea (OSA), particularly in the elderly. Metabolic syndrome (MetS) increases cardiovascular risk in the general population; however, less is known about its influence in patients with OSA. We aimed to assess whether MetS affected the risk of MACE and all-cause mortality in elderly patients with OSA.

**Methods:**

From January 2015 to October 2017, 1,157 patients with OSA, aged ≥60 years, no myocardial infarction (MI), and hospitalization for unstable angina or heart failure were enrolled at baseline and were followed up prospectively. OSA is defined as an apnea-hypopnea index of ≥5 events per hour, as recorded by polysomnography. Patients were classified on the basis of the presence of MetS, according to the definition of the National Cholesterol Education Program (NCEP). Incidence rates were expressed as cumulative incidence. Cox proportional hazards analysis was used to estimate the risk of all events. The primary outcomes were MACE, which included cardiovascular death, MI, and hospitalization for unstable angina or heart failure. Secondary outcomes were all-cause mortality, components of MACE, and a composite of all events.

**Results:**

MetS was present in 703 out of 1,157 (60.8%) elderly patients with OSA. During the median follow-up of 42 months, 119 (10.3%) patients experienced MACE. MetS conferred a cumulative incidence of MACE in elderly patients with OSA (log-rank, *P* < 0.001). In addition, there was a trend for MACE incidence risk to gradually increase in individuals with ≥3 MetS components (*P* = 0.045). Multivariate analysis showed that MetS was associated with an incidence risk for MACE [adjusted hazard ratio (aHR), 1.86; 95% confidence interval (CI), 1.17–2.96; *P* = 0.009], a composite of all events (aHR, 1.54; 95% CI, 1.03–2.32; *P* = 0.036), and hospitalization for unstable angina (aHR, 2.01; 95% CI, 1.04–3.90; *P* = 0.039). No significant differences in the risk of all-cause mortality and other components of MACE between patients with and without MetS (*P* > 0.05). Subgroup analysis demonstrated that males (aHR, 2.23; 95% CI, 1.28–3.91, *P* = 0.05), individuals aged <70 years (aHR, 2.36; 95% CI, 1.27–4.39, *P* = 0.006), overweight and obese individuals (aHR, 2.32; 95% CI, 1.34–4.01, *P* = 0.003), and those with moderate-severe OSA (aHR, 1.81;95% CI: 1.05–3.12, *P* = 0.032) and concomitant MetS were at a higher risk for MACE.

**Conclusion:**

MetS is common in elderly patients with OSA in the absence of MI, hospitalization for unstable angina or heart failure. Further, it confers an independent, increased risk of MACE, a composite of all events, and hospitalization for unstable angina. Overweight and obese males, aged <70 years with moderate-severe OSA combined with MetS presented a significantly higher MACE risk.

## Introduction

Obstructive sleep apnea (OSA) is the most common form of sleep-related breathing disorders (1). It has become a leading health concern owing to its growing prevalence and strong association with all-cause mortality (2–4). In addition to a higher risk of acute coronary syndrome (5), recent data demonstrate that OSA confers an increased risk of composite cardiovascular endpoints, including myocardial infarction (MI), hospitalization for heart failure, and cardiovascular death. These risks have not been sufficiently addressed with current OSA treatment strategies (6).

Understanding the link between OSA, long-term cardiovascular risks, and all-cause mortality are imperative for devising effective preventive strategies. OSA is often associated with cardiovascular disease risk factors, such as MetS, diabetes, hypertension, and obesity (7–9); therefore, it is possible that a convergence of multiple risk factors could potentiate cardiovascular risks and all-cause mortality. This is exemplified by MetS, which is a highly prevalent, multifaceted disease. It is characterized by a series of abnormalities that include abdominal adiposity, hypertension, dyslipidemia, and elevated fasting plasma glucose (10).

The growing burden of obesity, sedentary lifestyles, and dietary patterns has led to an increase in the prevalence of MetS. Further, it has been associated with a higher risk of MACE when compared with general population (11). Our group has previously shown a longitudinal association between type 2 diabetes and MACE, hospitalization for unstable angina, and a composite of all events in elderly patients with OSA (12). A recent study reported that prediabetes, the precursor stage of diabetes, is often accompanied with a manifestation of much broader underlying disorders, including MetS (13). However, the incidence and long-term risk of cardiovascular disease and all-cause mortality related to MetS in elderly patients with OSA have not been established. Furthermore, questions remain as to whether MetS confers an incremental risk of MACE or all-cause mortality, beyond the cumulative incidence tendency that is contributed by OSA itself. Resolving these issues is important before considering MetS as part of the preventive strategies in patients with OSA, especially in the elderly population.

We hypothesized that MetS confers a higher risk of long-term cardiovascular disease (CVD) and all-cause mortality in elderly patients with OSA. Hence, the primary aim of the present study was to assess the prognostic implication of MetS for incident MACE (cardiovascular death, MI, and hospitalization for unstable angina or heart failure) in a cohort of patients with OSA in the absence of MI, hospitalization for unstable angina, or heart failure at baseline. Secondary outcomes included the individual components of MACE, a composite of all events, and all-cause mortality.

## Methods

### Study Design and Participants

**Figure 1:**
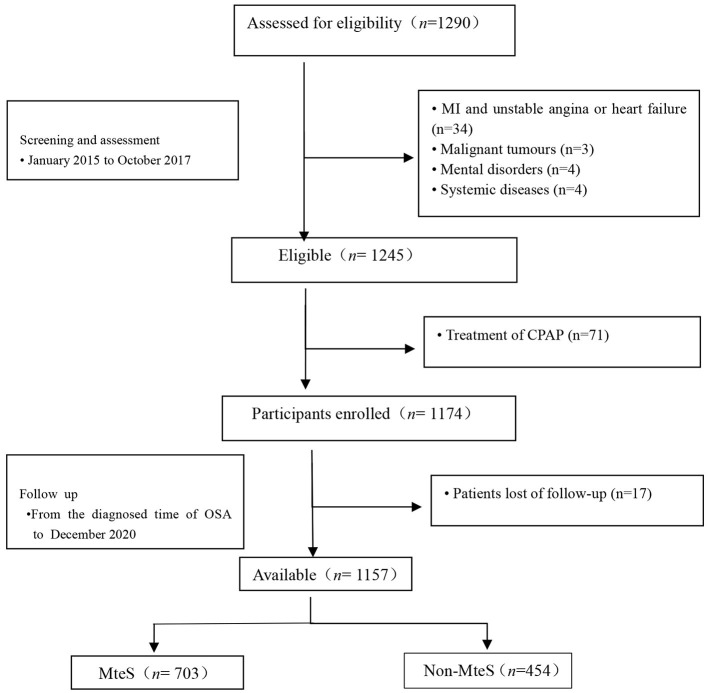
Study flowchart. MteS, metabolic sydrome; CPAP indicates continuous positive airway pressure; MI, myocardial infarction.

This study was designed as an multi-center, prospective, observational cohort study that recruited elderly (age ≥ 60 years) patients with OSA free of MI, hospitalization for unstable angina, or heart failure at baseline. This was diagnosed at the departments or sleep medicine centers of six hospitals, including Chinese PLA General Hospital, Peking University International Hospital, Peking University People's Hospital, Beijing Chaoyang Hospital, 960th Hospital of PLA, and the affiliated Hospital of Gansu University of Chinese Medicine between January 2015 to October 2017. OSA was defined as an apnea-hypopnea index (AHI) of ≥5 events per hour. The AHI was calculated as the number of apnea and hypopnea events per hour of sleep. Taken together, we consecutively enrolled 1,290 patients with a first diagnosis of OSA who underwent an overnight sleep study after clinical stabilization during hospitalization at the sleep center (within 1 week after admission) of six hospitals. The study flowchart is presented in Figure 1. Inclusion criteria were (1) aged ≥60 years and (2) a diagnosis of OSA. Exclusion criteria were (1) a diagnosis of MI, hospitalization for unstable angina, or heart failure; (2) history of malignant tumors; (3) mental disorders; (4) systemic diseases; and (5) previous OSA diagnosis or continuous positive air pressure (CPAP) treatment. Furthermore, we excluded those lost during follow-up. The final study population was 1,157 elderly patients with OSA.

This study conformed to the Strengthening the Reporting of Observational studies in Epidemiology (STROBE) guidelines. It was performed in accordance with the Declaration of Helsinki. The study was approved by the Ethics Committee of Chinese PLA General Hospital (S2019-352-01) and all participants provided a written informed consent.

### Overnight Sleep Study

All patients underwent an overnight sleep study within 1 week after admission at a sleep center (from 21:00 to 07:00 the next day). The sleep study was performed using a portable laboratory-based polysomnography (PSG) instrument (Compumedics, Melbourne, Australia), as described previously (12). Patients abstained from caffeine, hypnotic drugs, or sedatives for 1 day before their sleep study. OSA diagnosis and sleep tests were first scored according to the Guideline of the American Academy of Sleep Medicine (2012) (14). Standard PSG parameters were measured, including continuous polygraphic recordings from surface leads for electroencephalography, electrooculography, electrocardiography, nasal and oral airflow, thoracic and abdominal impedance belts for respiratory effort, pulse oximetry for oxyhemoglobin concentration, tracheal microphone for snoring, and a sensor for sleep position. Data were subjected to automatic computer analysis followed by manual correction by two sleep technologists and a senior physician. OSA was defined as AHI ≥5 events/hour. AHI was calculated as the total number of apnea and hypopnea events divided by the sleep duration (in hours). OSA was classified as mild (AHI = 5–14.9), moderate (AHI = 15–30), or severe (AHI >30) (14, 15).

### Covariates

Participant baseline characteristics were designated as regular laboratory test data from the 2nd day after the overnight study. These included demographic data [age, sex, body mass index (BMI), systolic blood pressure (SBP), diastolic blood pressure (DBP), waist-hip ratio, neck circumference, waist circumference, and self-reported smoking and alcohol use]; laboratory data [fasting plasma glucose (FPG), triglyceride (TG), high-density lipoprotein (HDL)]; comorbidities [diabetes, hypertension, hyperlipidemia, atrial fibrillation (AF), chronic obstructive pulmonary disease (COPD), carotid atherosclerosis, hyperlipidemia, coronary heart disease (CHD)]; and sleep parameters [oxygen desaturation index (ODI), mean pulse oxygen saturation (MSpO_2_), lowest pulse oxygen saturation (LSpO_2_), duration of time with SaO_2_ <90% (TSA90), percentage of times SaO_2_ <90% during the total monitoring time of overnight sleep (T90), and apnea time]. These data were collected by two experienced physicians who were blinded to the clinical outcomes and sleep patterns of the patients using pre-established case report forms. Disagreements were resolved by consensus. The categories of covariates were listed in Supplementary Table S1.

BMI was expressed in kg/m^2^. Smoking was defined as at least one cigarette per day currently or within the past 2 years. Drinking was defined as drinking once per week for at least half a year. SBP and DBP were measured three times. Hypertension was recorded if the mean of at least two consecutive measurements of SBP/DBP was ≥140/90 mmHg or the use of antihypertension medication (16). Dyslipidemia was defined using the Chinese Guidelines for the management of hyperlipidemia in adults. This was defined as 1) serum cholesterol concentration ≥4.7 mmol/L; 2) TG concentration ≥2.3 mmol/L; or 3) low-density lipoprotein concentration ≥4.1 mmol/L. Patients who met one of these three criteria were defined as having hyperlipidemia (17). AF was defined based on the ESC 2016 guidelines (18). Type 2 diabetes was defined as existing diabetes treatment or fasting blood glucose ≥7.0 mmol/L and 1) 2-h oral glucose tolerance test ≥11.1 mmol/L or 2) hemoglobin A_1C_ ≥6.5% (19). Carotid atherosclerosis, CHD, and COPD were determined using the records of relevant diagnostic clinical (Read) codes indicating the presence of the condition (20).

### MetS Assessment

MetS was defined according to the National Cholesterol Education Program Adult Treatment Panel III (NCEP ATP III) criteria (with the modified waist circumference criteria for Asians) as the presence of at least three of the following five clinical features: (1) waist circumference ≥80 cm in women, and ≥90 cm in men; (2) elevated plasma TG (≥1.7 mmol/L), or treatment for high TG; (3) low-plasma HDL (<1.3 mmol/L for women and <1.03 mmol/L for men); (4) high FPG (≥5.6 mmol/L) or currently taking anti-diabetic medication; and (5) SBP ≥130 or DBP ≥85 mmHg or current treatment for hypertension (21, 22).

### Procedures, Follow-Up, and Outcomes

Each patient was closely managed in the sleep centers of the six study hospitals according to the American Academy of Sleep Medicine guidelines on OSA (2012) (14). All patients underwent PSG within 7 days of admission and regular laboratory tests on the 2nd day after the overnight study. Patients with OSA (AHI ≥15 events/hour), particularly those with excessive daytime sleepiness, were referred to the sleep center for further evaluation.

Patients were prospectively followed-up for approximately 4 years after their diagnosis and PSG assessment. All follow-ups were completed by December 2020. Follow-ups ended at the first MACE or all-cause mortality. Patients or their proxies were contacted by telephone by two investigators who were blinded to patients' PSG results at 1 month, 3 months, 6 months, 1 year, and then every 6 months thereafter (at least 3 months and up to 1 years). Participant follow-up outcomes were further verified by a clinic visit and medical chart review, which lasted until end of the study. The cause of death was ascertained from hospital discharge letters or death certificates provided by patients' family members. In difficult cases, three senior investigators, blinded to MetS, status adjudicated study outcomes by a consensus of opinion. All clinical events were confirmed by source documentation and were adjudicated by the clinical event committee.

The primary endpoint of our study was MACE, defined as MI, cardiovascular death, and hospitalization for unstable angina or heart failure. Secondary outcomes were all-cause mortality, a composite of all events, and individual components of MACE.

### Statistical Analysis

Continuous variables are shown as mean ± SD or median (interquartile range). Categorical variables are shown as counts and proportions (%). The quantitative variables were not normally distributed; therefore, the Mann-Whitney U test was used for further analyses. The relationship between MetS and time-to-event End Points were summarized using Kaplan-Meier curves and compared using the log-rank test. Crude and adjusted hazard ratios (aHR), and their corresponding 95% confidence intervals (CI), were calculated for the association between MetS and incidence risk of all events using Cox proportional hazards regression models. Two Cox proportional hazards regression models were conducted to examine the association between OSA with MetS and long-term MACE risks, and all-cause mortality in elderly patients. Model 1 was unadjusted; Model 2 was adjusted for potential risk factors, including age, sex, BMI, alcohol use, SBP, DBP, waist circumference, WHR, neck circumference, FPG, TG, HDL, comorbidities of CHD, hyperlipidemia, hypertension, carotid atherosclerosis, diabetes, and sleep parameters of AHI, ODI, T90, TSA90, and LSpO_2_. A 2-sided *P* <0.05 was considered statistically significant. All analyses were conducted using the SPSS (version 25.0, SPSS Inc., Chicago, Illinois, USA).

## Results

### Baseline Characteristics

After excluding patients with MI, hospitalization for unstable angina, or heart failure at screening, 1,245 eligible patients were identified. Of these, 71 had previously received CPAP treatment. Therefore, 1,174 patients were enrolled. Follow-up status was unavailable for 17 (1.4%) patients. Finally, 1,157 patients with OSA were included in the analyses (Figure 1). Patient baseline characteristics (median age, 66.0 years, 60.8% male) are presented in Table 1. Among the 1,157 patients, 454 (39.2%) had no MetS (median age, 65 years, male/female = 263/191) and 703 (60.8%) had MetS (median age, 66 years, male/female = 441/262), with AHI values of 28.6 and 25.6, respectively (*P* = 0.004).

**Table 1:** General characteristics of study subjects according to MetS.

	**Total (*n* = 1,157)**	**Non-MteS (*n* = 454)**	**MteS (*n* = 703)**	***P*-value**
**Demographics**				
Age, y	66.0 (62.0, 71.0)	65.0 (62.0, 69.0)	66.0 (63.0, 72.0)	<0.001
Male, *n* (%)	704 (60.8)	263 (57.9)	441 (62.7)	0.102
BMI, kg/m^2^	26.4 (24.0, 29.0)	25.1 (22.8, 27.3)	27.2 (24.7, 29.7)	<0.001
NC, mm	38.0 (35.0, 40.0)	37.5 (35.0, 40.0)	38.0 (35.5, 41.0)	0.043
waist-hip ratio, %	90 (79, 102)	85 (75, 96)	93 (83, 105)	<0.001
Drinking, *n* (%)	116 (10.0)	36 (7.9)	80 (11.4)	0.055
Smoking, *n* (%)	256 (22.1)	97 (21.4)	159 (22.6)	0.608
SBP, mmHg	133.0 (124.0, 144.0)	124.0 (120.0, 128.0)	140.0 (133.0, 150.0)	<0.001
DBP, mmHg	76.0 (70.0, 81.0)	73.0 (70.0, 80.0)	78.0 (70.0, 85.0)	<0.001
WC, mm	91.0 (80.0, 100.0)	87.5 (78.0, 96.0)	94.0 (86.0, 102.0)	<0.001
FPG, mmol/L	5.7 (5.1, 6.6)	5.4 (5.0, 6.1)	5.9 (5.3, 6.8)	<0.001
TG, mmol/L	1.5 (1.0, 1.9)	1.3 (1.0, 1.7)	1.6 (1.1, 2.0)	<0.001
HDL, mmol/L	1.1 (0.9, 1.4)	1.2 (1.0, 1.5)	1.0 (0.9, 1.3)	<0.001
**Sleep parameters**				
AHI, events/h	27.2 (14.95, 45.40)	25.6 (13.9, 39.3)	28.6 (15.6, 48.4)	0.004
TST, h	7.05 (6.14, 7.47)	7.05 (6.10, 7.45)	7.04 (6.17, 7.47)	0.632
ODI, events/h	22.1 (10.3,40.5)	19.5 (10.1, 34.7)	23.5 (11.0, 43.2)	0.002
TSA90, min	14.00 (2.28, 60.52)	10.44 (1.82, 46.45)	16.45 (2.90, 69.25)	0.003
T90, %	3 (0, 15)	3 (0, 12)	4 (1, 17)	0.003
MSpO_2_, %	93.0 (91.7,95.0)	93.7 (92.0, 95.0)	93.0 (91.0, 95.0)	0.099
LSpO_2_, %	80.0 (72.0, 85.0)	81.0 (74.0, 85.0)	80.0 (71.0, 85.0)	0.033
Average heart Rate, events/min	63.0 (57.6, 68.4)	63.0 (58.0, 68.6)	63.5 (57.4, 68.3)	0.833
Average apnea time, s	22.4 (19.5, 25.4)	22.3 (19.1, 25.7)	22.4 (19.6, 25.3)	0.999
Maximum apnea time, s	51.0 (32.9, 75.0)	49.0 (32.0, 74.2)	51.7 (33.0, 75.0)	0.966
**Medical history**, ***n*** **(%)**				
Severity of OSA				0.035
Mild OSA	289 (25.0)	123 (27.1)	166 (23.6)	
Moderate OSA	347 (30.0)	148 (32.6)	199 (28.3)	
Severe OSA	521 (45.0)	183 (40.3)	338 (48.1)	
Hypertension	739 (63.9)	47 (10.4)	692 (98.4)	<0.001
CHD	265 (22.9)	62 (13.7)	203 (28.9)	<0.001
Hyperlipidemia	325 (28.1)	90 (19.8)	235 (33.4)	<0.001
AF	97 (8.4)	40 (8.8)	57 (8.1)	0.664
Carotid atherosclerosis	296 (25.6)	90 (19.8)	206 (29.3)	<0.001
Diabetes	286 (24.7)	54 (11.9)	232 (33.0)	<0.001
COPD	80 (6.9)	34 (7.5)	46 (6.5)	0.536

*BMI, body mass index; NC, neck circumference; WC, waist circumference; WHR, waist/hip ratio; SBP, systolic blood pressure; DBP, diastolic blood pressure; AHI, the apnea-hypopnea index; FPG, fasting plasmaglucose; TG, triglyceride; HDL, high-density lipoprotein; ODI, the oxygen desaturation index; MSpO_2_, the mean pulse oxygen saturation; LSpO_2_, the lowest pulse oxygen saturation; TSA90, the duration of time with SaO_2_ <90%; T90, percentage of the times for SaO_2_ <90% in total monitoring time during overnight sleep; LAT, the longest apnea time; MAT, the mean apnea time; OSA, obstructive sleep apnea; CHD, coronary heart disease; AF, atrial fibrillation; COPD, chronic obstructive pulmonary disease*.

Patients with MetS had a significantly higher proportion of comorbidities, such as hypertension (98.4 vs. 10.4%), CHD (28.9 vs. 13.7%), hyperlipidemia (33.4 vs. 19.8%), carotid atherosclerosis (29.3 vs. 19.8%), diabetes (33.0 vs. 11.9%) (all *P* <0.001). Additionally, their median age (66 vs. 65 years), BMI (27.2 vs. 25.1 kg/m^2^), waist-hip ratio (93 vs. 85%), SBP (140 vs. 124 mmHg), DBP (78 vs. 73 mmHg), neck circumference (38.0 vs. 37.5 mm) waist circumference (94.0 vs. 87.5 mm), FPG (5.9 vs. 5.4 mmol/L), TG (1.6 vs. 1.3 mmol/L), ODI (23.5 vs. 19.5 times/h), TSA90 (16.45 vs. 10.44 min), T90 (4 vs. 3%) were significantly higher (all *P* < 0.001). By contrast, patients without MetS were more likely to have a higher median HDL (1.2 vs. 1.0 mmol/L) and LSpO_2_ (81 vs. 80%) (all *P* < 0.001). Conversely, there were no differences with respect to sex, drinking status, smoking status, AF, COPD, and other sleep parameters (all *P* > 0.05) between patients with and without MetS.

### Primary Outcomes: MACE

**Figure 2:**
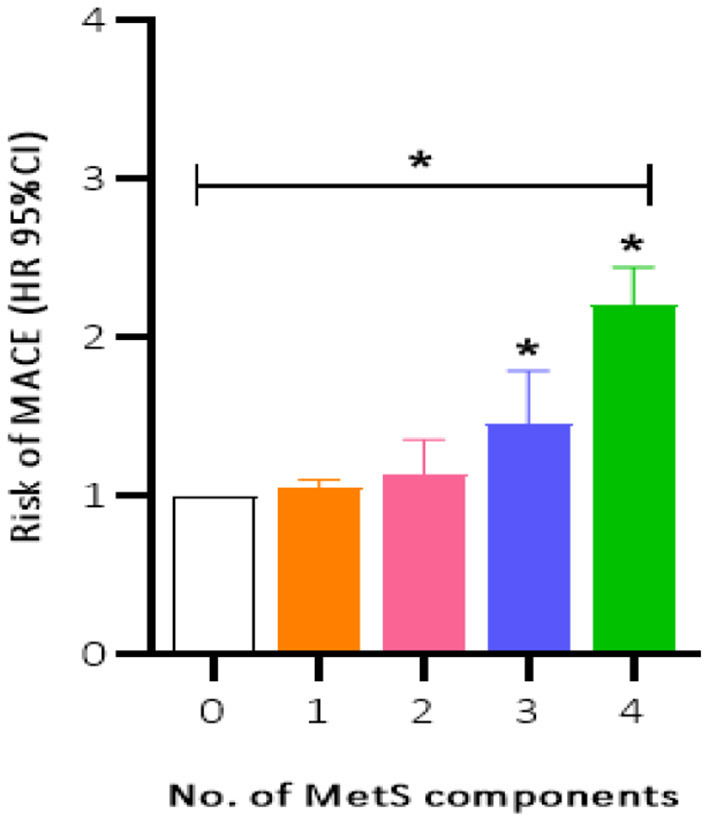
Incidence risk of MACE (HR 95% CI) by the number of the MetS components present in patients, *P* for trend = 0.045. MACE, major adverse cardiovascular event; MetS, metabolic syndrome. *P < 0.05.

**Figure 3:**
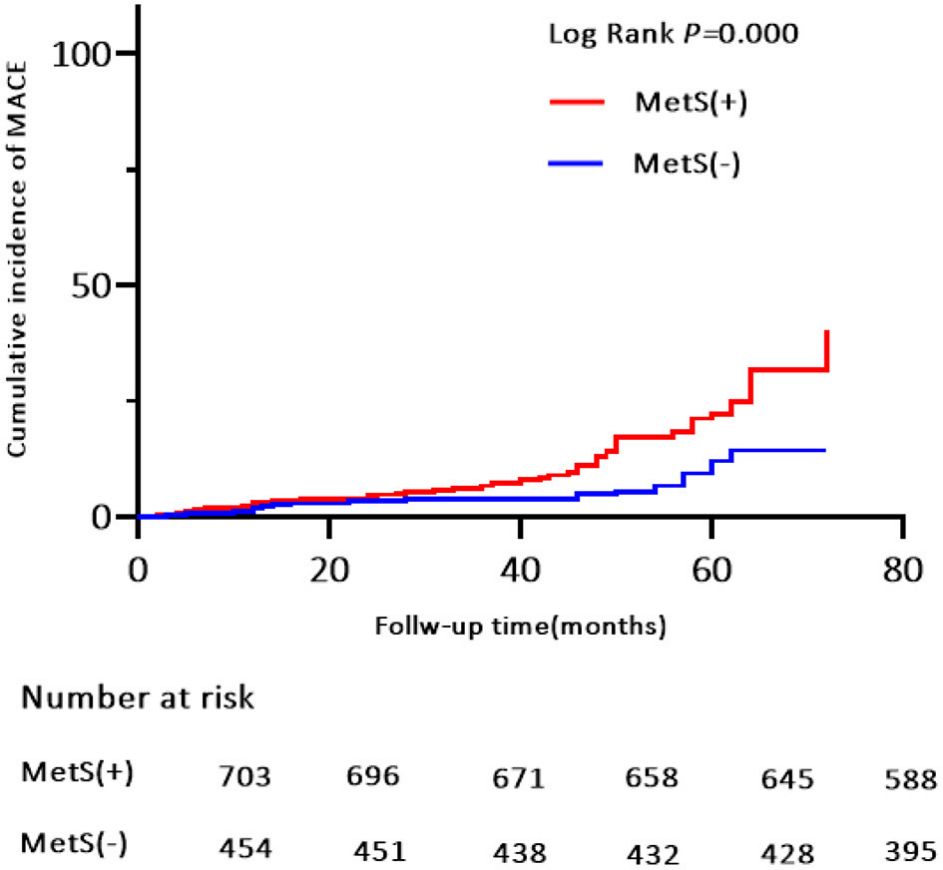
Kaplan-Meier estimates of cumulative incidence (%) for MACE (Primary end points). Log-rank test, *P* = 0.000. MACE, major adverse cardiovascular event.

Patients were prospectively followed for 42 months or until MACE, providing 1,157 patient observations. MACE occurred in 119 (10.3%) patients at a median follow-up period of 42 months (range, 1–72 months); 90 (12.8%) of those patients had MetS and 29 (6.4%) had no MetS. To further elucidate the relationship between MetS and MACE in elderly OSA patients, we analyzed the incidence risk of MACE (HR 95% CI) according to the number of MetS components in individual patients. There was a trend for MACE incidence risk to gradually increase in individuals with ≥3 MetS components (*P* = 0.045) (Figure 2). Additionally, the Kaplan-Meier curve that associated MetS status with the cumulative incidence of MACE demonstrated a significantly higher cumulative event-rate of MACE in patients with MetS when compared with patients without MetS (Log-rank test, *P* = 0.000; Figure 3).

The unadjusted Cox proportional hazard model showed that MetS was associated with a HR of 2.13 (95% CI, 1.36–3.34; *P* = 0.001) for MACE. After adjustment for age, sex, BMI, alcohol use, WHR, comorbidities of CHD, hyperlipidemia, hypertension, carotid atherosclerosis and diabetes, sleep parameters of AHI, ODI, T90, TSA90 and LSpO_2_, and MetS components, the HR for MACE was moderately attenuated; however, it remained statistically significant (aHR, 1.86; 95% CI, 1.17–2.96; *P* = 0.009; Table 2). In the subgroup analysis, the aHR for MACE by MetS were higher in overweight and obese individuals (aHR, 2.32; 95% CI, 1.34–4.01, *P* = 0.003), males (aHR, 2.23; 95% CI, 1.28–3.91, *P* = 0.005), those aged <70 years (aHR, 2.36; 95% CI, 1.27–4.39, *P* = 0.006), and patients with moderate-severe OSA (aHR, 1.81; 95% CI, 1.05–3.02, *P* = 0.032; Table 3).

**Table 2:** Association between MetS and incidence of all events.

	**Unadjusted analysis**		**Adjusted analysis**	
	***HR* (95*% CI*)**	***P*-Value**	***HR* (95*% CI*)**	***P*-Value**
MACE	2.13 (1.36, 3.34)	0.001	1.86 (1.17, 2.96)	0.009
Cardiovascular death	2.32 (0.92,5.82)	0.074	2.01 (0.74, 5.39)	0.173
MI	2.22 (1.07, 4.57)	0.031	1.66 (0.73, 3.79)	0.227
Hospitalization for unstable angina	2.58 (1.43, 4.67)	0.002	2.01 (1.04, 3.90)	0.039
Hospitalization for heart failure	3.11 (0.67, 14.34)	0.147	5.087 (0.59, 4.25)	0.141
All-cause mortality	1.86 (1.02 3.40)	0.042	1.56 (0.84, 2.83)	0.162
Composite of all events	2.04 (1.41, 2.95)	0.000	1.54 (1.03, 2.32)	0.036

*MACE, major adverse cardiovascular event; MI, myocardial infarction*.

**Table 3:** Subgroup analysis of the associations between MetS and MACE.

	**Unadjusted analysis**		**Adjusted analysis**	
	***HR* (95*%CI*)**	***P*-Value**	***HR* (95*%CI*)**	***P*-Value**
**Age**				
<70	2.66 (1.53,4.62)	0.001	2.36 (1.27, 4.39)	0.006
≥70	1.62 (0.85, 3.10)	0.143	1.48 (0.71, 3.08)	0.291
**Severity of OSA**				
Mild	2.66 (1.13,6.24)	0.025	1.92 (0.73, 5.04)	0.188
Moderate-severe	2.23 (1.37,3.61)	0.001	1.81 (1.05, 3.12)	0.032
**Gender**				
Male	2.40 (1.40,4.13)	0.002	2.23 (1.28, 3.91)	0.005
Female	2.14 (1.10,4.18)	0.026	1.7 3(0.86, 3.49)	0.125
**BMI**				
Normal (18.5–22.9)	1.14 (0.43,3.01)	0.785	0.68 (0.22, 2.12)	0.501
Overweight and obese (≥23)	2.84 (1.72,4.68)	0.000	2.32 (1.34, 4.01)	0.003

*BMI, body mass index; OSA, obstructive sleep apnea*.

### Secondary Outcomes: All-Cause Mortality, Components of MACE, and a Composite of All Events

The crude values for the secondary end points events are shown in Table 4. Fifty-four patients died during the follow-up period. The proportions of MetS vs. non-MetS patients was 5.5 vs. 3.3%. Kaplan-Meier analysis revealed significant differences in the incidence of all-cause mortality (Log-rank test, *P* = 0.038; Supplementary Figure S1). Similarly, univariate analysis showed that MetS was associated with a higher (approximately 4-year) risk of all-cause mortality in elderly OSA patients (HR, 1.86; 95% CI, 1.02–3.04, *P* = 0.042). However, the aHR for all-cause mortality fell short of statistical significance (aHR, 1.56; 95% CI, 0.84–2.83, *P* = 0.162; Table 2).

**Table 4:** Crude number of adverse events during follow-up.

**Follow-up outcomes**	**Total** **(*n* = 1,157)**	**MteS** **(*n* = 703)**	**Non-MteS** **(*n* = 454)**
MACE, *n* (%)	119 (10.3)	90 (12.8)	29 (6.4)
Cardiovascular death, *n* (%)	25 (2.2)	19 (2.7)	6 (1.3)
MI, *n* (%)	38 (3.3)	28 (4.0)	10 (2.2)
Hospitalization for unstable angina, *n* (%)	64 (5.5)	50 (7.1)	14 (3.1)
Hospitalization for heart failure, *n* (%)	11 (1.0)	9 (1.3)	2 (0.4)
All-cause mortality, *n* (%)	54 (4.7)	39 (5.5)	15 (3.3)
Composite of all events, *n* (%)	145 (12.5)	106 (15.1)	39 (8.6)

**Figure 4:**
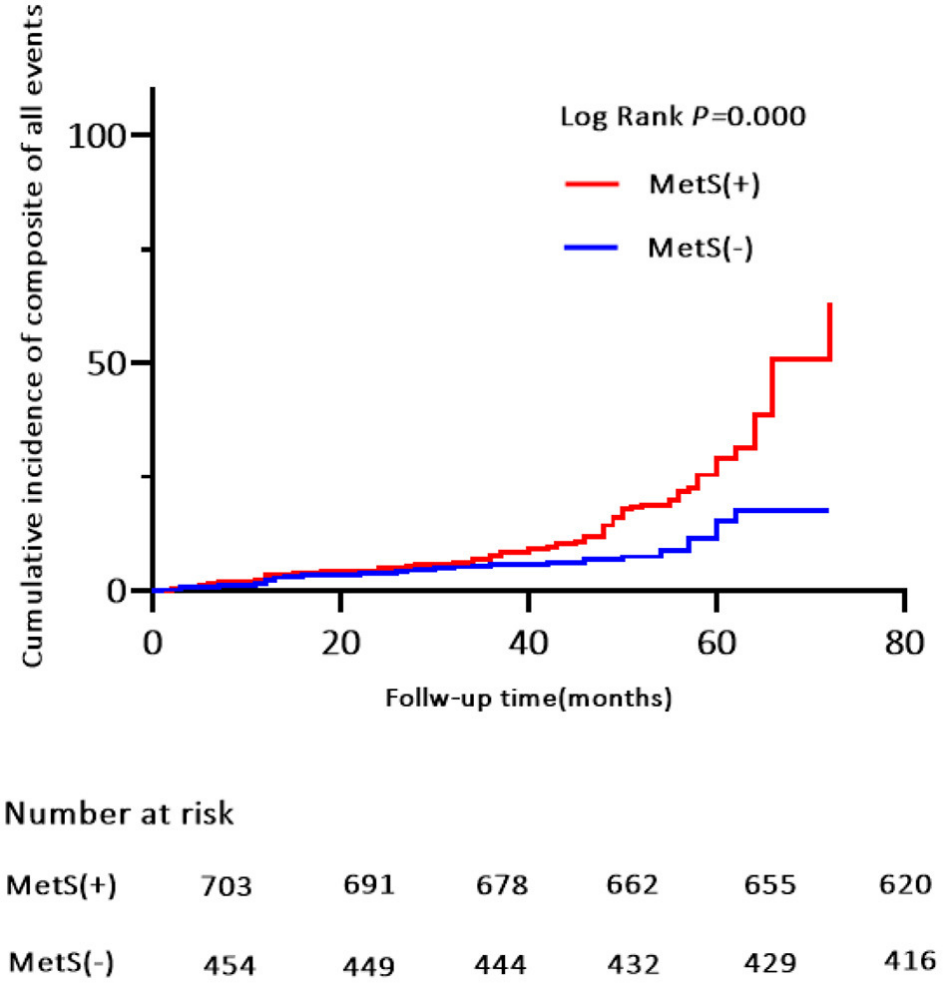
Kaplan-Meier estimates of cumulative incidence (%) for composite of all events. Log-rank test, *P* = 0.000.

**Figure 5:**
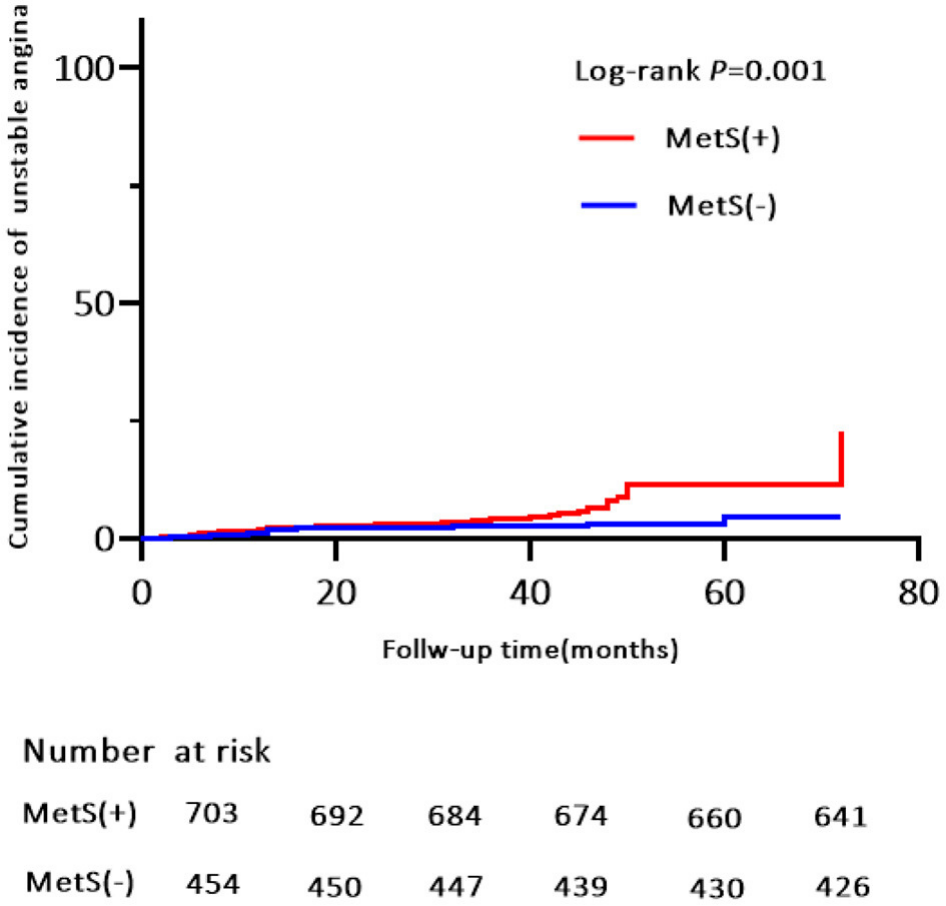
Kaplan-Meier estimates of cumulative incidence (%) for hospitalization for unstable anginaI. Log-rank test, *P* = 0.001.

Of the 119 MACE events, 38 (3.3%) patients had MI and 75 (6.5%) patients were hospitalized for heart disease (64 and 11 cases of hospitalization for unstable angina and heart failure, respectively). In addition, 25 (2.2%) patients died of a cardiovascular event during the follow-up period (Table 4). Kaplan-Meier analysis showed no significant differences in the incidence of cardiovascular death and hospitalization for heart failure, except for a higher rate of hospitalization for unstable angina, composite of all events, and MI in the MetS group than in the non-MetS group (log-rank test, *P* = 0.001, *P* = 0.000, *P* = 0.024, respectively, Figures 4, 5, Supplementary Figure S2). Multivariate analysis showed a higher risk of hospitalization for unstable angina in patients with MetS when compared with those without MetS (aHR, 2.01; 95% CI, 1.04–3.90; *P* = 0.039; Table 2). Moreover, the incidence of a composite of all events was significantly higher in the MetS group than in the non-MetS group in the adjusted Cox regression analysis (aHR, 1.54; 95% CI, 1.03–2.32; *P* = 0.036; Table 2).

## Discussion

This was a multi-center, prospective, observational study of 1,157 elderly patients with OSA in the absence of MI, hospitalization for unstable angina, or heart failure at baseline. First, we found that MetS, as defined by the NCEP ATP III criteria, was highly prevalent in our study population; it affected more than two thirds of the elderly patients with OSA. Second, patients with MetS had a higher incidence risk of MACE during the median 42-month follow-up when compared with patients without MetS. Moreover, the trend of MACE risk gradually increased in patients with ≥3 MetS components. Third, MetS conferred an approximately 2.0-fold higher risk for MACE, 1.54-fold risk for a composite of all events, and independently increased the risk of hospitalization for unstable angina. Finally, the risk of MACE was more strongly correlated with MetS in obese and overweight males aged <70 years and in patients with moderate-severe OSA.

Approximately 17% of the total population is affected by OSA; however, its prevalence varies widely according to age, ethnicity, and sex of the population studied (23). MetS is strongly associated with long-term cardiovascular risks, such as MI, hospitalization for heart failure or unstable angina, and cardiovascular death (24); therefore, this study is important to address the public health care burden of OSA-related complications. A cross-sectional study investigated the association between MetS and the attenuation of heart rate recovery after maximal exercise (ΔHRR) by grouping all participants into two categories (AHI ≥ 15 events/h or AHI <15 events/h) in the Brazilian population. They findings indicated that ΔHRR is impaired to a greater degree where OSA patients with MetS (25). Roche et al. used data from a population-based sample of older adult participants in South Africa and founded that the components of MetS (such as waist circumference, HDL, and TG, et al.) was associated with a increased risk of cardiometabolic risk (CMR) in elderly OSA patients (26). Moreover, another study demonstrated that OSA increases sympathetic peripheral and central chemoreflex response in young to middle-aged patients with MetS from the Unit of Cardiac Rehabilitation and Exercise Physiology of the Heart Institute (InCor), which seems to explain the increase in sympathetic nerve activity and consequent may cause a increased risk of CVD (27). Also, previous one epidemiological study among 1,727 Asian OSA patients (aged 30–54) in Shanghai showed that cardiometabolic disorders (including the biomarkers of MetS) in OSA may potentiate their unfavorable effects on CVD, which is in concordance with our results, but the study was a cross-sectional study and excluded a geriatric population (10). Our study population had a median age at mid-sixties, which emphasizes the awareness of the elderly at risk of MACE and early initiation of prevention strategies. Moreover, we found that individuals who exhibited three or more components of MetS had a gradual increase in the risk of MACE, indicating that more complex MetS phenotypes may lead to worsened prognoses. Therefore, it is crucial to minimize the Mets-related cardiovascular risk in patients with OSA.

Most MACE in our study presented as hospitalization for unstable angina (5.5%), which confirms observations that there was a higher risk for unstable angina than other individual components of MACE in patients with MetS during the median 42-month follow-up. OSA involves complex mechanisms, including mechanical, chemical, neural, hemodynamic, and inflammatory processes, that may interact to increase the risk of MACE (28, 29). OSA patients have a higher risk of MACE and all-cause mortality (4). A recent population-based cohort study has shown that OSA and OSA-induced hypoxia may correlate with the severity of MI, increase the incidence of heart rhythm disorders in elderly patients with subacute MI, and worsen their short-term poor outcomes (30). A growing amount of evidence has reported the risk factors for MetS, including obesity, poor diet, sedentary behavior, and genetics. These share considerable overlap with OSA risk factors; therefore, the severity of OSA may be related to MetS. The co-existence of MetS and OSA may aggravate the severity of carotid intima-media thickness and atherosclerosis. Additionally, it potentiates other underlying mechanisms of CVD, which may explain the increased incidence risk for MACE (30–32). Thus, our study further investigated the impact of concomitant MetS on the long-term risk of MACE in elderly patients with OSA. Notably, our findings as a multicenter OSA population-based study that adjusted for potential confounders confirmed the significance for MACE between patients with OSA with and without MetS.

MetS predisposes individuals to OSA development; epidemiological studies have reported that MetS is 6–9 times more likely to be present in individuals with OSA when compared with the general population (7). In our study, patients with MetS demonstrated risk factor clustering and a higher burden of comorbidities (e.g., carotid atherosclerosis, hypertension, and hyperlipidemia) pertinent to long-term prognosis. Of note, patients with MetS experienced MACE approximately 2-times more often when compared with patients without MetS. As a result, the median 42-month crude number of cardiovascular deaths, MI, hospitalization for unstable angina, and hospitalization for heart failure in patients with MetS (2.7, 4.0, 7.1, and 1.3%, respectively) was considerably higher than in patients free of MetS (1.3, 2.2, 3.1, and 0.4%, respectively). Several potential mechanisms the association between MetS and risks of MACE in elderly OSA patients. Firstly, this association may be related to metabolic changes that transpire during sleep in OSA. OSA recurrence increases plasma free fatty acids (FFAs) and glucose during sleep, associated with sympathetic and adrenocortical activation. Recurring exposure to these metabolic changes may foster CVD risks (33). Secondly, the increased expression of mineralocorticoid receptor (MR) in the elderly, which may provided a further explanation for enhanced cardiovascular risk in the elderly. MR activation contributes to increase blood pressure with aging by vascular oxidative stress that are important mechanisms of CVD risk in OSA and MetS. In the elderly, dysregulation of MR signaling is associated with increased cardiovascular risk in OSA patients combined MetS (34, 35). Thirdly, preclinical and clinical trials have demonstrated that GLP-1 receptors are abundantly present in the heart (36). GLP-1 receptor agonists (GLP-1RAs), a group of widely used anti-hyperglycaemic agents, which work on the incretin axis and improve insulin secretion, has resulted in improved cardiovascular outcomes along with improved metabolic control and significant weight reduction (37).

To the best of our knowledge, this is the first study to report MetS as a multivariable predictor of MACE in elderly patients with OSA. A previous study has shown that the prevalence of MetS declines in older age and that the cardiometabolic comorbidities associated with OSA diminish in parallel (38). Additional studies have demonstrated that the incidence of stroke, but not coronary heart disease, is increased in elderly patients with severe OSA (39). Our data further demonstrated that MetS diagnosed in patients aged <70 years with moderate-severe OSA have a higher risk (approximately 4-year) of MACE. This may be explained by the fact that the prevalence of OSA peaks at <70 years of age. Furthermore, moderate intermittent hypoxia can protect the myocardium from ischemic injury in elderly patients with mild OSA (40).

There are higher risk-adjusted odds of survival in obese and overweight patients with heart failure (41), which is consistent with the “obesity paradox”. However, recent evidence has shown a shift in the obesity paradox with aging; there are diminished cardiac benefits with overweight and obesity in elderly patients (42). Obesity is a common pathogenic factor of OSA and MetS. Our datas revealed that MetS was associated with a higher incidence risk of MACE in overweight and obese elderly patients with OSA. Nonetheless, further research is needed to clarify the underlying mechanisms and to define the optimal incidence risk for MACE to reduce CVD complications in obese and overweight OSApatients combined MetS.

OSA and MetS lead to CVD (4). The prevalence of MetS and OSA are different in males and females. A cross sectional study has shown that women with OSA have higher chances of having MetS than men (43). Our findings revealed that male elderly patients with OSA and MetS had a higher risk of MACE. This may be due to metabolic differences, which are more prevalent in women following hormonal changes like menopause. This may explain the delay in OSA or MetS peak prevalence when compared with men. In addition, our study showed that patients with MetS were associated an increased incidence of all-cause mortality; however, this was not significant. This may be because the patients with OSA and Mets in our study were under guideline-based therapy and in a stable condition with no target organ damage. Nonetheless, the complex pathophysiology of MetS in elderly patients with OSA and the potential impact on all-cause mortality and MACE cannot be ignored in clinical risk assessment, diagnosis, and treatment.

## Study Limitations

First, a major limitation to the present study is the homogenous Asian study population. This means that the study's findings are not representative of the global population and should be evaluated in other ethnicities. Second, MetS status and its components are variable; they can change dynamically over time. This could have biased the results. Third, the incidence of MACE and all-cause mortality are complex processes and correlated with multiple factors. We adjusted for as many CVD-related risk factors as possible; however, there may be other unmeasured confounders. Nevertheless, these limitations do not fundamentally affect the value of our study.

## Conclusion

In our Asian population-based multicenter cohort study, MetS was a complex risk factor that independently increased the risk for MACE, hospitalization for unstable angina, and a composite of all events in elderly patients with OSA in the absence of MI, hospitalization for unstable angina, or heart failure. In the subgroup analysis, overweight and obese males, aged <70 years, with moderate-severe OSA concomitant MetS presented a higher risk for MACE. Our findings reinforce the clinical utility of MetS for CVD risk assessment in elderly patients with OSA. Furthermore, we suggest that MetS is considered as a modifiable risk factor in cardiovascular prevention in patients with OSA. This possibility requires further confirmation in future large-scale, multi-racial, multicenter, prospective cohort studies.

## Data Availability Statement

The original contributions presented in the study are included in the article/Supplementary Material, further inquiries can be directed to the corresponding authors.

## Ethics Statement

The studies involving human participants were reviewed and approved by the Ethics Committee of Chinese PLA General Hospital (S2019-352-01). The patients/participants provided their written informed consent to participate in this study.

## Author Contributions

LL, XS, ZZ, JH, JLi, WX, ZH, YiG, YaG, KC, JG, LZ, and HW collected the data. LL, XS, and ZZ analyzed the data and wrote the manuscript draft. JH revised the manuscript. JLin, TL, and XF designed this study. All authors have read and approved the manuscript.

## Conflict of Interest

The authors declare that the research was conducted in the absence of any commercial or financial relationships that could be construed as a potential conflict of interest.

## Publisher's Note

All claims expressed in this article are solely those of the authors and do not necessarily represent those of their affiliated organizations, or those of the publisher, the editors and the reviewers. Any product that may be evaluated in this article, or claim that may be made by its manufacturer, is not guaranteed or endorsed by the publisher.

## Abbreviations

CVD: Cardiovascular disease
MetS: Metabolic syndrome
OSA: Obstructive sleep apnoea
MACE: Major adverse cardiovascular events
PSG: Polysomnography
AHI: The apnoea-hypopnoea index
BMI: Body mass index
NC: Neck circumference
WC: Waist circumference
WHR: Waist/hip ratio
SBP: Systolic blood pressure
DBP: Diastolic blood pressure
TG: Triglyceride
HDL: High-density lipoprotein
FPG: Fasting plasma glucose
LAT: The longest apnea time
MAT: The mean apnea time
TOT: Total monitoring time
T90: Percentage of the times for SaO_2_ <90% in TOT during overnight sleep
TSA90: The duration of time with SaO_2_ <90%
ODI: The oxygen desaturation index
MSpO_2_: The mean pulse oxygen saturation
LSpO_2_: The lowest pulse oxygen saturation
CHD: Coronary heart disease
COPD: Chronic obstructive pulmonary disease
MI: Myocardial infarction
AF: Atrial fibrillation
CPAP: Continuous positive airway pressure
aHR: Adjusted hazard ratios
CI: Confidence intervals
NCEP: National cholesterol education program
ATP: Adult treatment panel.
