# Supplementary material for: Association of Metabolic Syndrome With Long-Term Cardiovascular Risks and All-Cause Mortality in Elderly Patients With Obstructive Sleep Apnea
Source: Front Cardiovasc Med. 2022 Feb 7;8:813280. doi: 10.3389/fcvm.2021.813280 (PMC8859338; doi:10.3389/fcvm.2021.813280)
Supplement: Supplementary file 1 [file Data_Sheet_1.docx]

**Association of metabolic syndrome with long‐term cardiovascular risks and all-cause mortality in elderly patientswith obstructive sleep apnea**

Supplementary Table S-1 Characterics of covariates

| Covariates | Category | Definition |
| --- | --- | --- |
| Sex, n (%) | Categorical variables | Male, female |
| Age, year | Continuous variable |  |
| BMI, kg/m^2^ | Continuous variable |  |
| SBP, mmHg | Continuous variable |  |
| DBP, mmHg | Continuous variable |  |
| Current smoker, n (%) | Categorical variables | Never smoking, Ever or Current smoking |
| Current drinker , n (%) | Categorical variables | Never alcohol drinking, Ever or Current alcohol drinking |
| FPG, mmol/L | Continuous variable |  |
| TG, mmol/L | Continuous variable |  |
| Waist circumference, cm | Continuous variable |  |
| neck circumference, cm | Continuous variable |  |
| Waist-hip ratio | Continuous variable |  |
| HDL, mmol/L | Continuous variable |  |
| LAT, s | Continuous variable |  |
| MAT, s | Continuous variable |  |
| T90, % | Continuous variable |  |
| TST, h | Continuous variable |  |
| AHI, events/h | Continuous variable |  |
| ODI, events/h | Continuous variable |  |
| MSpO_2_, % | Continuous variables |  |
| LSpO_2_, % | Continuous variables |  |
| TSA90, min | Continuous variables |  |
| CHD, n (%) | Categorical variables | Yes, No |
| Hyperlipidemia, n (%) | Categorical variables | Yes, No |
| Hypertension, n (%) | Categorical variables | Yes, No |
| AF, n (%) | Categorical variables | Yes, No |
| Carotid atherosclerosis, n (%) | Categorical variables | Yes, No |
| COPD, n (%) | Categorical variables | Yes, No |
| Diabetes | Categorical variables | Yes, No |

BMI: body mass index; NC: neck circumference; WC: waist circumference;WHR: waist/hip ratio; SBP: systolic blood pressure; DBP: diastolic  blood pressure; AHI: the apnea-hypopnea index; FPG: fasting plasmaglucose; TG: triglyceride; HDL: high*-*density lipoprotein; ODI: the oxygen desaturation index; MSpO_2_: the mean pulse oxygen saturation; LSpO_2_: the lowest pulse oxygen saturation; TSA90: the duration of time with SaO_2_<90%;T90: percentage of the times for SaO_2_＜90% in total monitoring time during overnight sleep; LAT: the longest apnea time; MAT: the mean apnea time; OSA: obstructive sleep apnea; CHD: coronary heart disease; AF:atrial fibrillation; COPD: chronic obstructive pulmonary disease.


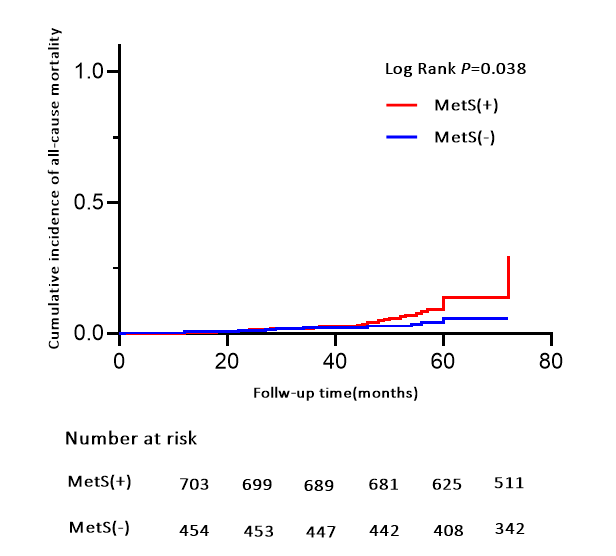


Figure S-1. Kaplan-Meier estimates of cumulative incidence (%) for all-cause mortality. Log-rank test:*P*=0.038.


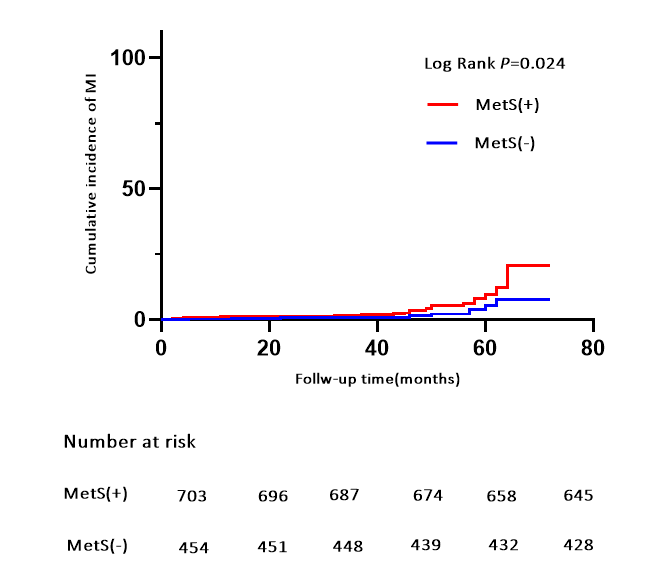


Figure S-2. Kaplan-Meier estimates of cumulative incidence (%) for MI. Log-rank test: *P*=0.024.

MI: myocardial infarction.
